# Bacterial diversity in saliva and oral health-related conditions: the Hisayama Study

**DOI:** 10.1038/srep22164

**Published:** 2016-02-24

**Authors:** Toru Takeshita, Shinya Kageyama, Michiko Furuta, Hidenori Tsuboi, Kenji Takeuchi, Yukie Shibata, Yoshihiro Shimazaki, Sumio Akifusa, Toshiharu Ninomiya, Yutaka Kiyohara, Yoshihisa Yamashita

**Affiliations:** 1 Section of Preventive and Public Health Dentistry, Division of Oral Health, Growth and Development, Kyushu University Faculty of Dental Science, Fukuoka, Japan; 2 Department of Preventive Dentistry and Dental Public Health, School of Dentistry, Aichi-Gakuin University, Nagoya, Japan; 3 Department of Oral Health Management, School of Oral Health Science, Kyushu Dental University, Kitakyushu, Japan; 4 Division of Research Management, Center for Cohort Studies, Graduate School of Medial Sciences, Kyushu University, Fukuoka, Japan; 5 Department of Environmental Medicine, Graduate School of Medical Sciences, Kyushu University, Fukuoka, Japan

## Abstract

This population-based study determined the salivary microbiota composition of 2,343 adult residents of Hisayama town, Japan, using 16S rRNA gene next-generation high-throughput sequencing. Of 550 identified species-level operational taxonomic units (OTUs), 72 were common, in ≥75% of all individuals, as well as in ≥75% of the individuals in the lowest quintile of phylogenetic diversity (PD). These “core” OTUs constituted 90.9 ± 6.1% of each microbiome. The relative abundance profiles of 22 of the core OTUs with mean relative abundances ≥1% were stratified into community type I and community type II by partitioning around medoids clustering. Multiple regression analysis revealed that a lower PD was associated with better conditions for oral health, including a lower plaque index, absence of decayed teeth, less gingival bleeding, shallower periodontal pockets and not smoking, and was also associated with tooth loss. By contrast, multiple Poisson regression analysis demonstrated that community type II, as characterized by a higher ratio of the nine dominant core OTUs, including *Neisseria flavescens*, was implicated in younger age, lower body mass index, fewer teeth with caries experience, and not smoking. Our large-scale data analyses reveal variation in the salivary microbiome among Japanese adults and oral health-related conditions associated with the salivary microbiome.

The human oral cavity is colonized by numerous and diverse microorganisms as commensals. These bacteria constitute complex microbial communities on intraoral surfaces, and dental plaque microbiota that form on the teeth are the cause of two major oral diseases, dental caries and periodontitis. Mutans streptococci are the major etiologic agent of dental caries^1^ and *Porphyromonas gingivalis, Tannerella forsythia* and *Treponema denticola* are prime suspects in periodontitis^2^. Furthermore, recent studies that have used open-ended molecular approaches and the 16S rRNA gene have implicated other commensal members with the etiology of each disease, such as lactobacilli for dental caries^3^ and as many as 17 species, including *Filifactor alosis* for periodontitis^4^.

Saliva is a biological fluid secreted from the salivary glands into the oral cavity and contains bacteria shed from adhering microbial communities on various intraoral surfaces, including tooth surfaces, gingival crevices, tongue dorsum, and buccal mucosa. Oral bacteria in a planktonic state (as in saliva) are not generally regarded as direct causal agents of the oral diseases. However, intraoral transmission of pathogenic bacteria is likely to be mediated by bacteria dispersed via saliva^5^,^6^.

The salivary microbiome, which is comprised of indigenous bacteria that are specific to each person, exhibits long-term stability (on the scale of years)^7^,^8^,^9^,^10^. On the other hand, oral disorders alter the structure of the teeth and their surroundings. Along with the loss of teeth, tooth decay and its treatment alter the structure of the tooth surfaces on which bacteria are attached. Gingival crevices around the teeth supply blood into the oral cavity when gingival inflammation occurs. Alveolar bone loss caused by periodontitis deepens the gingival crevices and creates an anaerobic niche (i.e., periodontal pockets). These ecological changes may affect the bacterial assemblage in saliva. The oral cavity is exposed to the external environment; therefore, the community structure may also be influenced by external factors such as smoking and personal oral hygiene. Furthermore, the host’s systemic condition (e,g., obesity) is reportedly associated with the microbiota structure in saliva^11^,^12^.

Based on these potential connections with the host’s health status, the salivary microbiome is promising as a surrogate indicator for health monitoring and disease diagnosis^13^. However, the degree of variation in the salivary microbiome at the population level has not been well characterized, although a “normal” community structure observed in healthy individuals has been demonstrated^14^,^15^,^16^,^17^. Furthermore, oral health-related conditions themselves often interact with each other; therefore, confounding effects should be taken into consideration for an accurate understanding of the relationship with the salivary microbiome. A large-scale comprehensive analysis of the salivary microbiome obtained from individuals with various health conditions is therefore required.

In this work, we collected saliva from Japanese adults inhabiting the town of Hisayama, which is recognized to be demographically representative of Japan^18^. The bacterial composition of saliva from more than 2,000 individuals was characterized using 16S rRNA gene next-generation sequencing, and we investigated the relationship with oral health-related conditions using statistical analyses via multivariate approaches. This population-based study of the salivary microbiome aimed to phylogenetically define commonly shared as well as uncommon taxa in saliva using *in silico* approaches, to reveal the variation in the salivary microbiome among Japanese adults and to investigate the oral health-related conditions associated with a bacterial assemblage in saliva.

## Results

We determined the bacterial compositions in the saliva of 2,343 adults aged ≥40 years living in Hisayama, Japan, using 16S rRNA gene amplicon analysis with an Ion PGM. In total, 67,753,985 reads were obtained from 14 sequencing runs, of which 32,855,304 quality-passed reads (14,022 ± 3,313 read per sample) containing the V1-V2 regions of bacterial 16S rRNA gene were used in the analyses. The sequences were assigned to 550 operational taxonomic units (OTUs) using a cutoff distance of 0.04. The rarefaction curve for the number of observed OTUs per sample almost reached a plateau after 5,000 sequence reads (Fig. S1).

### Community structure of salivary microbiome

**Figure 1:**
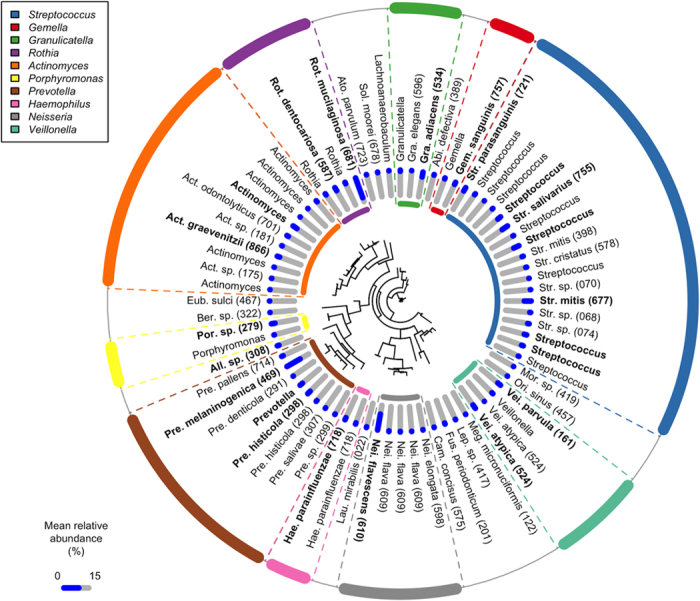
The 72 operational taxonomic units (OTUs) commonly (≥75%) identified in saliva of the individuals in the lowest quintile of phylogenetic diversity. The OTUs are ordered according to a phylogenetic tree built based on the 16S rRNA gene sequences using FastTree^50^; the tree is described in the center of the diagram. Mean relative abundance of each OTU in the individuals in the lowest PD quintile is shown as a blue bar. Of the 72 OTUs, 22 with a mean relative abundances of ≥1% in the saliva of individuals in the lowest PD quintile were shown in bold. Abbreviations: Mor; *Moraxella*; Ori, *Oribacterium*; Meg, *Megasphaera*; Lep, *Leptotorichia*; Fus, *Fusobacterium*; Cam, *Campylobacter*; Lau, *Lautropia*; All, *Alloprevotella*; Ber; *Bergeyella*; Eub, *Eubacterium*; Ato, *Atopobium*; Sol, *Solobacterium*; Abi, *Abiotrophia*.

**Figure 2:**
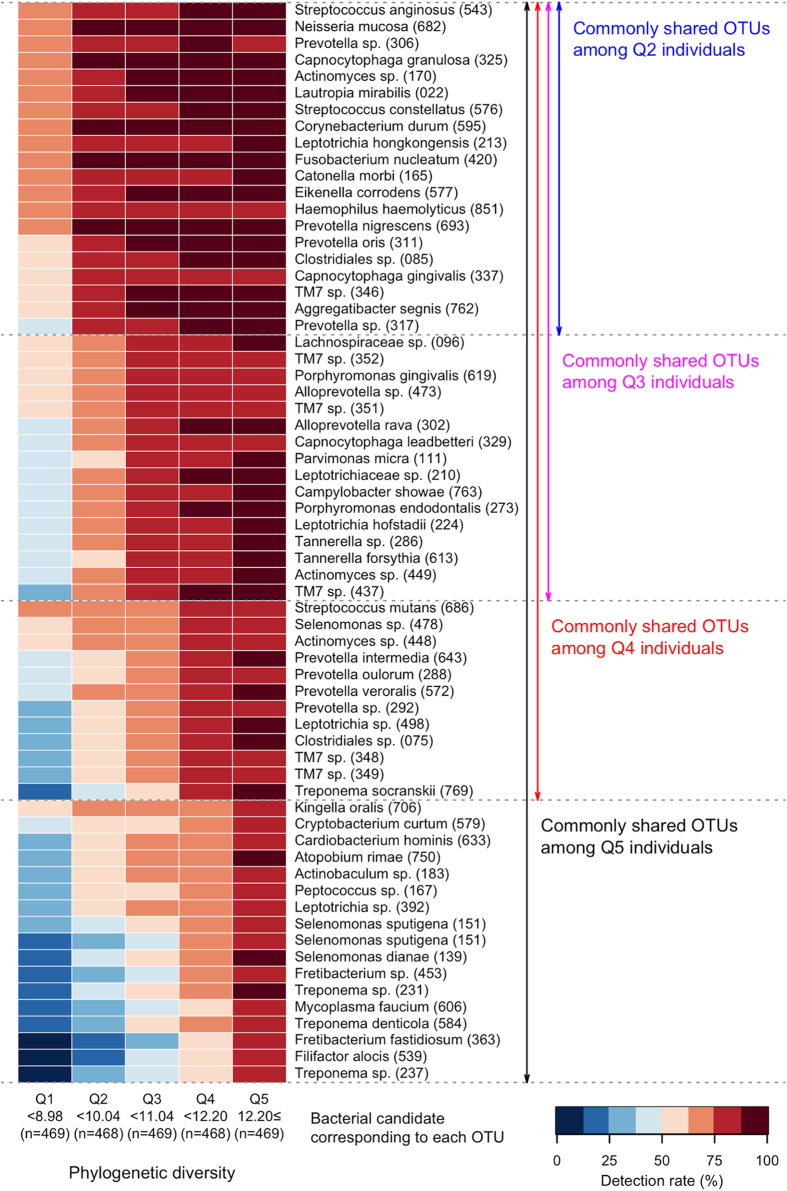
OTUs commonly (≥75%) identified in the saliva of the individuals in the highest quintile (Q5) of phylogenetic diversity, except for the 72 core OTUs shown in Fig. 1 . Of the 77 OTUs, only 65 corresponding to the bacterial species in the Human Oral Microbiome Database^49^ with a high (≥98%) identity are shown. OTUs shared among the lower quintile (Q2, Q3, Q4 and Q5) are shown separately. The detection rate (%) of each OTU in each quintile is shown in each grid by the color intensity.

We first calculated phylogenetic diversity (PD)^19^ to characterize the salivary bacterial populations. PD is an alpha diversity measure of microbial richness, which takes into account phylogenetic differences among species. The PD of the salivary microbiome in this study ranged from 2.74 to 17.57. We classified all 2,343 individuals into quintile categories (Q1, Q2, Q3, Q4 and Q5). The PD range for each quintile was as follows; PD < 8.98 for Q1, 8.98 ≤ PD < 10.04 for Q2, 10.04 ≤ PD < 11.04 for Q3, 11.04 ≤ PD < 12.20 for Q4 and 12.20 ≤ PD for Q5. As shown in Fig. 1, 72 OTUs were commonly (≥75%) present in the saliva of the 2,343 individuals, as well as those in every quintile category, including Q1. These “core” OTUs constituted the vast majority of the salivary microbiome in each individual (90.9 ± 6.1%, mean ± SD). This result suggests that the oral microbiome has a large set of bacterial taxa shared among individuals, consistent with the Human Microbiome Project (HMP) data^20^. These core OTUs corresponded to bacterial species such as *Streptococcus mitis, Streptococcus salivarius, Granulicatella adiacens, Neisseria flavescens, Rothia mucilaginosa* and *Prevotella melaninogenica* (Fig. 1). A higher PD value implies the presence of a broader array of bacterial species in the saliva. The number of commonly shared OTUs increased gradually in individuals with a higher PD quintile, as shown in Fig. 2. In total, 25 OTUs along with the 72 core OTUs were commonly (≥75%) found in the saliva of individuals in Q2. They corresponded to the bacterial species including *Fusobacterium nucleatum*, which is known as a middle colonizer in dental plaque development^21^. Furthermore, 18, 16 and 18 additional OTUs were identified in the individuals in Q3, Q4, and Q5, respectively. These OTUs corresponded to bacterial species, including the well-known periodontal pathogens *Porphyromonas gingivalis* (Q3), *Tannerella forsythia* (Q3), *Prevotella intermedia* (Q4), *Treponema denticola* (Q5), and *Filifactor alocis* (Q5), as well as cariogenic pathogens, including *Streptococcus mutans* (Q4).

### Phylogenetic diversity and oral health-related conditions

**Figure 3:**
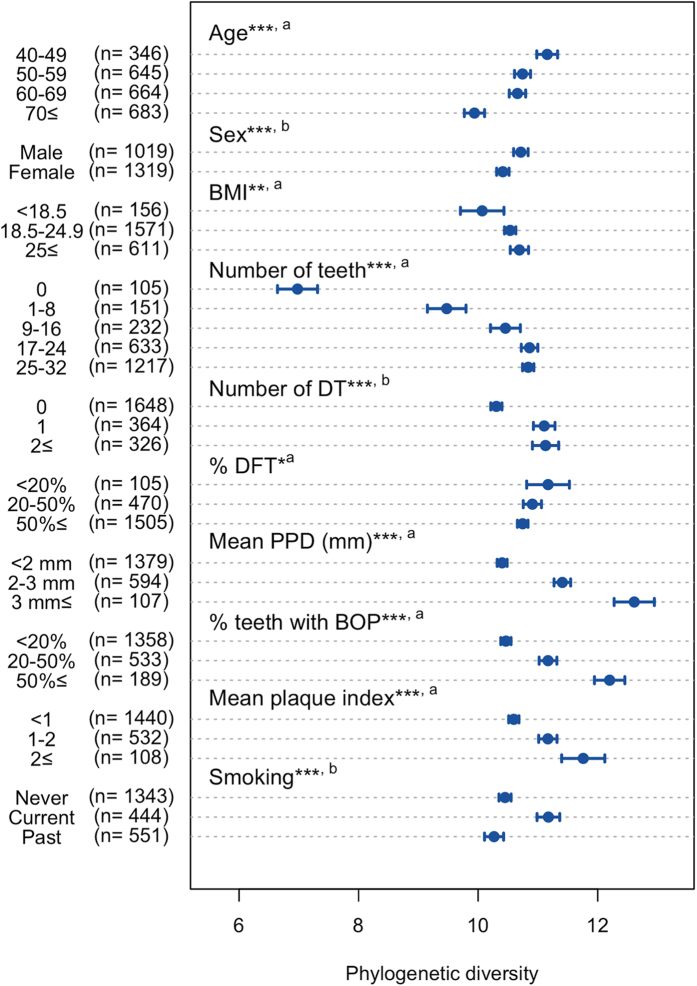
Phylogenetic diversity of salivary bacterial populations of the individuals for various general and clinical conditions. Of 2,343 individuals whose salivary bacterial composition was determined, 5 are excluded because of antibiotics use. Furthermore, 256 individuals with ≤8 teeth and 2 individuals from whom periodontal data were missing are excluded in %DFT, mean PPD, %teeth with BOP and mean plaque index. Dots indicate the mean; the error bars indicate 95% confidence intervals. ***P < 0.001, **P < 0.01, *P < 0.05 in bivariate analyses using Pearson’s correlation test^a^ or the Student’s *t*-test^b^. The individuals are categorized into two groups by the Student’s *t*-test in terms of the number of DT and smoking history (presence *vs*. absence and current smokers *vs*. the other individuals, respectively). Abbreviation: BMI, body mass index; DT, decayed teeth; DFT, decayed and filled teeth, PPD, periodontal pocket depth; BOP, bleeding on probing.

The PD of the salivary bacterial populations was significantly correlated with all oral health-related conditions evaluated in this study, as shown in Fig. 3. A bivariate analysis was used to extract these data; the PD values were higher in younger individuals, in males, in those with a higher body mass index (BMI), with more present teeth, who presented decayed teeth, with less teeth with caries experience, with deepened periodontal pockets, with a greater number of sites with bleeding on probing (BOP), with a higher plaque index, and in current smokers. The results were consistent when we used other alpha diversity indices, including the number of identified OTUs and Shannon diversity index (data not shown). We then performed multivariate regression analysis, incorporating the abovementioned variables to control for the effects of potential confounders (see Table 1). The results reveal that current oral conditions such as present teeth, decayed teeth, periodontal pockets, gingival bleeding and oral hygiene, along with current smoking, were significantly associated with the PD of the salivary bacterial populations, and that this was independent of other variables.

### Community type stratification of salivary microbiome

**Figure 4:**
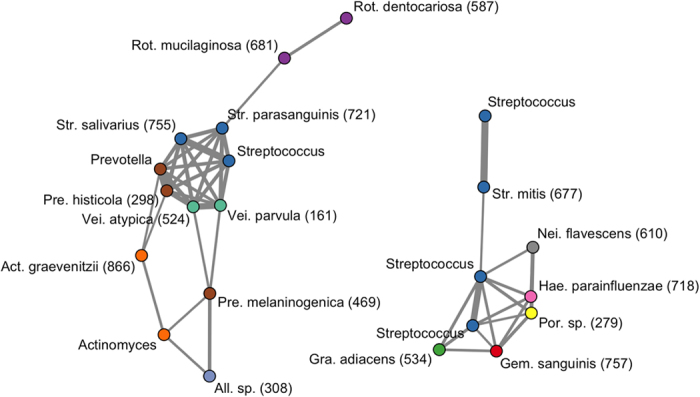
Microbial co-occurrence in the salivary bacterial population. Co-occurrence networks were constructed based on the relative abundances of 22 of 72 core OTUs shown in Fig. 1. The nodes represent the OTU; the nodes with the same color are classified as the same genus. Each edge represents a positive correlation between the two OTUs with an adjusted *P* value < 10^−12^ in Pearson’s correlation test, and their thickness corresponds to the coefficient values.

The variation in PD found here most likely depended on phylogenetic lineages of minority members of the microbiota, because the predominant members were mostly shared across individuals. We then assessed the relative abundances of common predominant bacteria to evaluate the salivary bacterial populations. Of the 72 core OTUs shown in Fig. 1, the 22 OTUs shown in bold (corresponding to bacterial species, including *N. flavescens, R. mucilaginosa*, and *S. salivarius*) had a mean relative abundances of ≥1% in the saliva of individuals in the lowest PD quintile. We focused on these “predominant core” OTUs, which accounted for 67.3 ± 8.8% of the salivary bacterial population in the participants overall. A co-occurrence network analysis based on the relative abundances suggested the presence of two cohabiting groups of bacteria: one was mainly composed of a bacterial complex including *Prevotella histicola, Veillonella parvula, Veillonella atypica, S. salivarius*, and *Streptococcus parasanguinis* (cohabiting group I), whereas the other was primarily assembled from *N. flavescens, Haemophilus parainfluenzae, Porphyromonas* sp. oral taxon 279, *Gemella sanguinis*, and *Granulicatella adiacens* (cohabiting group II, Fig. 4).

**Figure 5:**
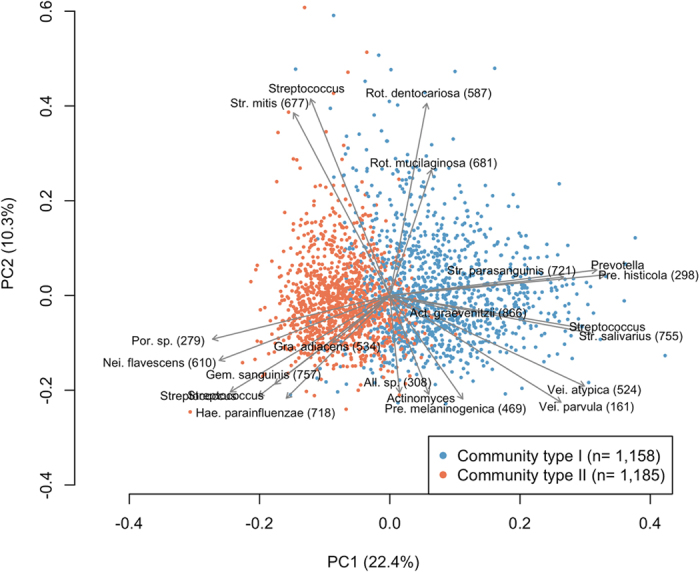
A principal component analysis biplot diagram showing similarity relationships among 2,343 relative abundance profiles for the 22 OTUs. The relative abundance profiles belonging to each community type are depicted using different colors. The direction and length of the arrows indicate how each OTU contributes to the first two components in the biplot. These two components explained 22.4% and 10.3% of the variance, respectively.

Partitioning around medoids (PAM) cluster analysis using the Jensen-Shannon divergence (JSD) and Calinski-Harabasz (CH) index were used for enterotype classification^22^. This allowed us to divide the relative abundance profiles into two. Figure 5 shows a PCA biplot diagram revealing the localization of the salivary bacterial populations belonging to each community type (represented as dots) in the negative and positive directions of the first principal component. The loading plot in the diagram (shown with arrows) indicates the bacterial composition of each community type: type I was characterized by the dominance of cohabiting bacterial group I (i.e., *Prevotella, Veillonella, Actinomyces, Rothia, S. salivarius*, and *S. parasanguinis*), whereas type II was characterized by the dominance of cohabiting bacterial group II (i.e., *Neisseiria, Haemophilus, Porphyromonas, Gemella*, and *S. mitis*). Table S1 lists the mean relative abundance of each OTU in the two community types. No significant difference was observed in terms of PD between the salivary bacterial populations belonging to each community type (10.4 ± 2.1 in community type I and 10.5 ± 1.7 in community type II).

### Community types and oral health-related conditions

Significant differences in general and clinical conditions were observed between individuals with type I and type II communities (Table 2). Individuals with a type II community exhibited a younger age, lower BMI, more present teeth, fewer teeth with caries experience, shallower periodontal pockets, fewer sites of BOP, and a lower plaque index; this group also included fewer individuals with decayed teeth and fewer current smokers than those with a type I community. We then performed multivariate Poisson regression analysis incorporating the abovementioned variables to control for the effects of potential confounding factors, allowing us to calculate odds ratios for the type I community (Table 3). The results reveal that age, BMI, dental caries experience, and current smoking are significantly associated with the community type in saliva, independent of other variables.

## Discussion

This population-based study determined the microbiota composition in the saliva of 2,343 Japanese adults inhabiting Hisayama, Japan, using a 16S rRNA gene amplicon deep sequencing approach. Hisayama is recognized to be demographically representative of Japan in terms of its age and occupational distributions, based on national census data^18^, and the population of this study constituted over half of the residents aged ≥40 years, including healthy individuals as well as those in various disease states. Although geographical differences in oral microbiome might exist between different regions of Japan, as observed between different countries^16^,^23^,^24^, our large-scale data are assumed to cover the variation in salivary microbiome among Japanese adults.

In the present study, 72 species-level OTUs were commonly present in the saliva of ≥75% of the individuals in the lowest PD quintile (i.e., Q1; Fig. 1). Commonly shared taxa (i.e., core microbiome) in saliva have been found previously^14^,^15^,^16^,^17^; however, their populations were mostly composed of healthy individuals because they aimed to define a “normal” oral microbiome. In contrast, those bacterial species identified here were shared among the least diverse microbiome in the community-dwelling population with various health conditions; thus, they could be regarded as a minimum set of the salivary microbiome in Japanese adults.

In general, good oral health was associated with a lower PD of the salivary microbiome, although an especially low PD was observed with high tooth loss (Fig. 3). Of 105 edentulous individuals, 94 belonged to Q1 and the mean PD of their microbiome (6.97) was far below the upper limit of the quintile (8.98). The salivary microbiome is a mixture of bacterial communities that exist at various sites in the oral cavity, although its community composition is most similar to the tongue microbiota^10^,^25^,^26^. The loss of bacterial communities associated with the tooth surface would therefore lead to a marked decrease in taxonomic richness in saliva. In dentate individuals, larger quantities of dental plaque and related deterioration in dental health were associated with greater PD of the microbiome. Prolonged plaque accumulation results in the multiplication of attached bacteria, as well as a compositional shift to a highly diverse community due to the altered ecological conditions within the biofilm^27^,^28^. Gingival bleeding provides a nutrient source, and deepened periodontal pockets provide an anaerobic niche in the gingival sulcus; both of these effects are associated with elevated plaque microbiota diversity^29^,^30^. Dental caries results in a roughened tooth surface to which bacteria can easily adhere, as well as an acidic microenvironment in the biofilm, which promotes the preferential growth of acid-tolerant bacteria in the plaque microbiota^31^. Furthermore, considering that the commonly shared taxa expanded in individuals with a higher PD quintile included periodontal and cariogenic pathogens acting within the plaque biofilm (e.g., *P. gingivalis* and *S. mutans*; Fig. 2), it is reasonable to expect that the PD of the salivary microbiome, which indicates the diversity of non-core minority bacteria, is affected by tooth-associated communities shed into saliva.

Although a bivariate analysis showed that age, gender, and BMI were significantly associated with PD, these relationships dissipated following multivariate adjustment (Table 1). Lower PD in older individuals is likely due to a decrease in the number of remaining teeth with age. Periodontal disease is more prevalent in males than in females^32^,^33^ and a high BMI is known to be associated with periodontitis^34^. A higher PD in male and obese individuals may be caused by the confounding effect of periodontal disease. We suggest that the PD of the salivary microbiome reflects local environmental conditions within the oral cavity, rather than inherent or systemic conditions of the host.

The salivary microbiome of Japanese adults could be categorized into two community types when we focused on the predominant core OTUs. The relative abundance data across the individuals shown in Fig. 4 suggest two cohabiting bacterial groups in saliva, and the ratio of these groups differed between the two types (Fig. 5 and Table S1). Based on 16S rRNA fingerprinting, we previously classified the salivary bacterial compositions of 200 individuals aged 15–40 years into three types: *Prevotella*/*Veillonella*-dominant type, *Streptococcus*-dominant type, and *Neisseria, Haemophilus*, or *Aggregatibacter*/*Porphyromonas*-dominant type^35^. A recent study using 16S rRNA deep sequencing also divided the relative abundances of common bacterial genera in the saliva of 161 healthy individuals into three types: *Prevotella*-dominant type, *Streptococcus*/*Gemella*-dominant type and *Neisseria*/*Fusobacterium*-dominant type^15^. Although the *Prevotella* and *Streptococcus* types were combined in the grouping used here based on the OTU abundances, the cohabiting bacterial groups observed in this study (Fig. 4) were consistent with previous results^15^,^35^.

Individuals with the microbiome categorized as community type II were significantly younger than those with the community type I microbiome (Table 2), and these differences were confirmed following multivariate adjustment (Table 3). Succession of oral microbiota has been observed in childhood^36^,^37^; however, little is known about the effects of aging on the microbiota in adults. Although a study using 16S rRNA deep sequencing indicated that the microbiota structure varied among different age groups, including adults^38^, there remains the possibility that it merely reflected their oral hygiene or health status. Our data are supported by statistical analyses and demonstrate that the relative abundances of predominant core bacteria in saliva are affected by aging, independently of other intraoral conditions.

The community type II salivary microbiome was also associated with a lower BMI after controlling for confounding effects (Tables 2 and 3). A Danish study reported that BMI had no statistically significant influence on the bacterial profiles of saliva^39^, and the association between obesity and the oral microbiome remains controversial. However, the microarray technique used in that study provided information only on bacterial taxa with a corresponding probe, and the relative abundance in total microbiota was not discussed. Our results for relative bacterial abundance are not inconsistent with their data; however, the lean and obese patterns shown here are not necessarily consistent with previous results linking oral health to obesity^11^,^12^. In particular, an Italian study^12^ found that Prevotellaceae and Veillonellaceae were less abundant in obese individuals, in contrast to our data (although the levels of obesity differed between the two studies). Further careful consideration, including other potential factors such as dietary habitat is required to determine the influence of obesity on the oral microbiome.

The dominant source of the salivary microbiome is most likely bacterial communities on the mucosal surfaces, especially the tongue dorsum, considering the similarities among microbiota compositions at various oral sites and in saliva^9^,^10^,^25^,^26^. Therefore, it is reasonable to expect that the relative abundances of predominant bacteria in saliva are not directly associated with the quantity of dental plaque or dental health (Table 3). Community type I was significantly correlated with higher caries experience, even following multivariate adjustment; it was not correlated with the presence of decayed teeth (Table 3). These community types might reflect susceptibility to dental caries in each individual, rather than the current condition. Another possibility is that the bacterial members that make up community type I simply prefer the environment created by dental restorations such as resin and metal. A longitudinal survey would clarify the role of the community type I microbiome in the development of dental caries.

Smokers have been reported to possess a more highly diverse, pathogen-rich, anaerobic subgingival plaque microbiota than non-smokers^40^. Greater PD in the salivary microbiome of current smokers (Fig. 3 and Table 1) would reflect the taxonomic richness of bacteria shed from the altered plaque microbiota. This work further demonstrates that smoking was significantly associated with salivary community type (Tables 2 and 3). We suggest that smoking has an impact not only on tooth-associated communities, but also on the mucosal microbiota in the oral cavity.

The ambiguous border between community types I and II in the PCA plot (Fig. 5) and the low mean silhouette width (0.19) of the PAM cluster suggest that the relative abundance profiles across Japanese adults should be continuous rather than binomial. The community types found in this study should be regarded as a stratification of the individuals for the investigation of oral health-related factors associated with a bacterial assemblage in saliva, rather than discrete patterns of the salivary microbiome.

The dominant core taxa identified in this study, including *N. flavescens, R. mucilaginosa, P. melaninogenica*, and *S. mitis* were also commonly present in saliva in results that analyzed US HMP data^41^. Our previous studies also demonstrated that dominant common members of salivary microbiome were common between Japanese and Koreans, although significant differences were observed in their relative abundances^24^. These results suggest that several bacterial taxa of the salivary microbiome might be shared among ethnically diverse individuals. Further cross-national studies are needed to clarify oral bacterial taxa shared globally and the effects of ethnic differences on oral microbiomes.

This study focused on the community structure of the salivary microbiome associated with oral health-related factors. However, no information on its metabolic capacity and microbial activity was given by 16S data, and thus it remains unclear how the structural differences affect the oral or systemic health in each individual. Future studies using metagenomic and transcriptomic approaches will help to clarify differences in the function of the salivary microbiome with different PD or community types and provide deeper insights into its role in human health.

This large-scale population-based study defined commonly shared and uncommon taxa in saliva, and revealed variation in the salivary microbiome among Japanese adults. Furthermore, statistical analyses using multivariate approaches identified oral health-related factors that were directly associated with each of the predominant and minority members. Our results suggest the utility of the salivary microbiome for evaluating the oral environment, and provide a basis for the development of an effective approach to maintain a healthy salivary microbiome.

## Methods

### Ethics statement

All participants understood the nature of the study and provided informed consent. The Ethics Committee of Kyushu University, Fukuoka, Japan approved the study design as well as the procedure for obtaining informed consent (reference numbers: 19B-1 and 26-312). All experiments were performed in accordance with the approved guidelines.

### Study population

Saliva samples were collected from participants of the Hisayama cohort study in Japan. A prospective population-based follow-up study of cardiovascular disease has been ongoing since 1961 in the town of Hisayama, which is a suburb of the Fukuoka metropolitan area in western Japan; the population of the town is approximately 8,000. As part of the follow-up survey, we performed a health examination of Hisayama residents in 2007, including a dental examination. Among all residents aged 40 years and older (4,298 individuals), 3,237 residents consented to participate in the study. Dental and medical examinations were performed on 2,930 individuals (68.2%). The subjects from whom sufficient saliva was not collected or from whom PCR amplicons appropriate for sequencing were not obtained were excluded. Thus, in total, 2,344 individuals (1,022 males, 1,322 females) took part in this study.

### Dental examination

The numbers of present, decayed or filled teeth were examined, as well as the use of dentures. The number of decayed and filled teeth signifies teeth with caries experience, and represents the caries history of the individual. Periodontal condition was evaluated based on the periodontal pocket depth (PPD) and bleeding on probing (BOP) at two sites for all teeth (mesio- and mid-buccal sites) based on the NHANES III method, with the exception of the third molars since, when partially impacted, these teeth frequently exhibit pseudopockets. Details of the periodontal examination are described elsewhere^42^. The periodontal pocket is the pathological space between the gingiva and the tooth root, and its depth is used in the clinical diagnosis of periodontal disease. The depth of these spaces is normally 1–3 mm in periodontally healthy individuals; however, it deepens as supporting connective tissue and alveolar bone become damaged by persistent gingival inflammation^43^. BOP is the bleeding caused by picking the inside of the periodontal pocket using a periodontal pocket probe, and is a visible symptom of gingival inflammation, which is suggestive of an active phase of periodontal disease. The oral hygiene status was assessed based on the dental plaque score according to the Silness and Löe plaque index^44^, which evaluates both soft debris and mineralized deposits on six selected teeth (positions 16, 12, 24, 36, 32, and 44). A score of 0–3 was given for each tooth, and the mean plaque score was calculated as an oral hygiene index for each individual. The participants were categorized into never, past, and current smokers based on interview data.

### Saliva collection and DNA extraction

Following the dental examination, the subjects were asked to chew gum for 2 min, and stimulated saliva samples were collected in sterile plastic tubes. The samples were stored at −30 °C until further analysis. DNA extraction from saliva samples was performed as described previously^45^.

### Ion Torrent 16S rRNA gene analysis

The 16S rRNA gene sequencing analysis was performed using saliva samples collected from the participants. The V1–V2 regions of 16S rRNA genes from each sample were amplified using the following primers: 8F (5′-AGA GTT TGA TYM TGG CTC AG-3′) with the adaptor A (5′-CCA TCT CAT CCC TGC GTG TCT CCG ACT CAG-3′) and the sample-specific 6-to-8-base tag sequence and 338R (5′-TGC TGC CTC CCG TAG GAG T-3′) with the Ion Torrent trP1 adaptor sequence (5′-CCT CTC TAT GGG CAG TCG GTG AT-3′). PCR amplification was carried out using KOD DNA polymerase (Toyobo, Osaka, Japan) under the following cycling conditions: 98 °C for 2 min followed by 30 cycles of 98 °C for 15 s, 60 °C for 20 s and 74 °C for 30 s. The amplicons were purified using an Agencourt AMPure XP Kit (Beckman Coulter, Brea, CA, USA) according to the manufacturer’s instructions. The DNA concentration and quality were assessed using a NanoDrop spectrophotometer (NanoDrop Technologies, Wilmington, DE, USA), and equal amounts of DNA were pooled together (up to 192 samples per pool). The pooled DNA was gel-purified using a Wizard SV Gel and PCR Clean-Up System (Promega, Madison, WI, USA), and the quality and fragment size were assessed using an Agilent 2100 Bioanalyzer (Agilent Technologies, Palo Alto, CA, USA). The DNA concentration was determined using a KAPA Library Quantification Kit (KAPA Biosystems, Wilmington, MA, USA), where the library pool was diluted to 13 pM for use as template DNA for emulsion PCR. Emulsion PCR and enrichment of template-positive particles were performed using an Ion PGM Template OT2 400 Kit (Life Technologies, Carlsbad, USA) and the Ion OneTouch 2 system (Life Technologies) according to the manufacturer’s instructions. The enriched particles were loaded onto an Ion 318 chip v2 (13 chips) or Ion 316 chip v2 (one chip) (Life Technologies), and sequencing was performed on an Ion PGM (Life Technologies) using an Ion PGM Sequencing 400 Kit (Life Technologies) according to the manufacturer’s instructions.

### Data analysis and taxonomy assignment

The raw sequence reads were trimmed using the CLC Genomics Workbench 6.5.1 (CLC Bio, USA, Cambridge, MA, USA) with a quality score limit of 0.05 and no ambiguous nucleotides. Reads were excluded from the analysis using a script written in R (version 3.0.1) if they were ≤200 bases (not including the tag sequence), had an average quality score ≤25, did not include the correct forward primer sequence or had a homopolymer run >6 nt. The remaining reads were assigned to the appropriate sample by examining the tag sequence. Quality-checked reads with the correct reverse primer sequence were dereplicated, and singleton reads were subsequently discarded. Similar sequences were clustered into OTUs using the –cluster_otus and –cluster_smallmem commands in UPARSE^46^, with a minimum pair-wise identity of 96%. All quality-checked reads were mapped to each OTU using the –usearch_global command in UPARSE by searching for OTU representative sequences. Chimeras were removed from the representative set after being identified using Chimera Slayer^47^ in QIIME^48^. One individual was excluded from the analysis because fewer than 5,000 quality-filtered reads were obtained from this individual. The taxonomy of representative sequences was determined using BLAST search against 831 oral bacterial 16S rRNA gene sequences (HOMD 16S rRNA RefSeq version 13.2) in the Human Oral Microbiome Database^49^ (Oral taxon IDs were given in parentheses following bacterial names in Figures). Nearest-neighbor species with ≥98% identity were selected as candidates for each representative OTU. The taxonomy of sequences with no BLAST hit was further determined using the RDP classifier with a minimum support threshold of 80% and the RDP taxonomic nomenclature (to the genus level). Rarefaction curves for the number of observed OTUs per sample were drawn using the rarecurve function in the vegan library of R. Following rarefaction to 5,000 reads per sample, the number of OTUs and the Shannon diversity index were calculated using the diversity function in the vegan library of R. A relaxed neighbor-joining tree was built using FastTree^50^, and phylogenetic diversity (i.e., the sum of all branch lengths in a 16S rRNA gene phylogenetic tree for each sample)^19^ was calculated using the pd function in the picante library of R. Bacterial community types were identified as described previously^22^; details of the procedure are described at http://enterotype.embl.de/enterotypes.html. Samples were clustered based on the relative abundances of predominant OTUs using JSD distance metric, and the PAM clustering algorithm using the pam function in the cluster library of R. The number of clusters and quality of the resulting clusters were chosen by maximizing CH index and the mean silhouette width using the index.G1 function in the clusterSim library of R.

### Statistical analysis

All statistical analyses were conducted using R (version 3.0.1). A principal component analysis (PCA) of the relative abundances of predominant OTUs was implemented using the dudi.pca function in the ade4 library. Student’s *t*-test was used to examine binomial data and Pearson’s correlation coefficient was used for continuous data; we investigated the relationship between each oral health-related factor and phylogenetic diversity. Multiple regression analyses were performed to investigate the multivariate associations between phylogenetic diversity and oral ecological factors using the lm function in the stats library. A Pearson correlation test followed by a false discovery rate correction was used to evaluate co-occurrence of dominant core OTUs. Co-occurrence networks were constructed using the gplot function in the sna library. Fisher’s exact test was used to examine binomial data and Student’s *t*-test was used for continuous data; we investigated the relationship between each oral health-related factor and community types. A multiple Poisson regression analysis was carried out to investigate the relationship between community types and oral ecological factors using the glm function in the stats library, following binomial logistic regression.

## Additional Information

**Accession codes:** The obtained sequence data were deposited in DDBJ Sequence Read Archive under BioProject ID PRJDB4107 (DRA003814, DRA003868–DRA003870, DRA003901– DRA003904, DRA004076–DRA004081).

**How to cite this article**: Takeshita, T. *et al*. Bacterial diversity in saliva and oral health-related conditions: the Hisayama Study. *Sci. Rep*. **6**, 22164; doi: 10.1038/srep22164 (2016).

## Supplementary Material

Supplementary Information

## Figures and Tables

**Table 1:** The relationship between phylogenetic diversity and general and clinical conditions.

	Standardized *β*	*P*value
Age	–0.013	0.55
Sex (male *vs*. female)	–0.035	0.10
BMI	0.024	0.20
Number of teeth	0.181	<0.001
Dental caries status		
Presence of DT	0.083	<0.001
Fraction of DFT (%)	–0.029	0.19
Periodontal conditions		
Mean PPD	0.234	<0.001
Fraction of teeth with BOP (%)	0.204	<0.001
Mean plaque index	0.071	0.004
Current smoking	0.124	<0.001

Adjusted R^2^ = 0.21.

A multivariate regression analysis was used to examine the association between environmental conditions and phylogenetic diversity of salivary microbiome of individuals with ≥9 teeth (n = 2,080).

Abbreviations: BMI, body mass index; DT, decayed teeth; DFT, decayed and filled teeth; PPD, periodontal pocket depth; BOP, bleeding on probing.

**Table 2:** General and clinical condition of individuals belonging to each community type.

	Community type		*P* value
	Type I	Type II	
	(n = 1,154)	(n = 1,184)	
Age, years	64.7 ± 11.8	60.2 ± 10.8	<0.001^a^
Sex, number of females (%)	661 (57.2)	658 (55.5)	0.42^b^
BMI	23.4 ± 3.5	22.9 ± 3.3	<0.001^a^
Number of teeth	19.8 ± 8.8	23.4 ± 7.2	<0.001^a^
Dental caries status			
Presence of DT, number (%)	377 (32.6)	313 (26.4)	0.001^b^
% DFT	70.1 ± 22.9	57.0 ± 22.8	<0.001^a^
Periodontal conditions			
Mean PPD	1.93 ± 0.67	1.74 ± 0.59	<0.001^a^
% teeth with BOP	20.9 ± 21.2	16.3 ± 18.6	<0.001^a^
Mean plaque index	0.77 ± 0.64	0.59 ± 0.56	<0.001^a^
Current smoking, number (%)	293 (25.3)	151 (12.7)	<0.001^b^

Of all 2,343 individuals whose salivary bacterial composition was determined, 5 were excluded because of antibiotics use. Additionally, 256 individuals with ≤8 teeth and 2 individuals for whom periodontal data were missing are excluded from the % DFT, mean PPD, % teeth with BOP and mean plaque index. ^a^Student’s *t*-test. ^b^Fisher’s exact test.

Values with errors are means ± standard deviation.

Abbreviations: BMI; body mass index; DT, decayed teeth; DFT, decayed and filled teeth; PPD, periodontal pocket depth; BOP, bleeding on probing.

**Table 3:** The relationship between community type and general as well as clinical conditions.

	Odds ratio	
	Crude (95% CI)	Adjusted (95% CI)
Age	1.01 (1.00–1.02)***	1.00 (1.00–1.01)**
Sex (male *vs*. female)	1.00 (0.88–1.14)	–
BMI	1.02 (1.01–1.04)**	1.03 (1.01–1.05)**
Number of teeth	0.96 (0.95–0.97)***	0.99 (0.98–1.00)
Dental caries status		
Presence of DT	1.19 (1.05–1.36)**	1.09 (0.95–1.25)
Fraction of DFT (%)	1.01 (1.01–1.02)***	1.01 (1.01–1.02)***
Periodontal conditions		
Mean PPD	1.27 (1.16–1.39)***	1.08 (0.96–1.22)
Fraction of teeth with BOP (%)	1.00 (1.00–1.00)***	0.99 (0.99–1.00)
Mean plaque index	1.27 (1.16–1.40)***	1.04 (0.92–1.18)
Current smoking	1.50 (1.30–1.73)***	1.70 (1.46–1.98)***

Dependent variable: community type (1: Community type I, 0: Community type II).

Bivariate and Poisson logistic regression analysis was used to examine the association between environmental conditions and community types of salivary microbiome of subjects with ≥ 9 teeth (n = 2,080). Crude and adjusted odds ratio were calculated by bivariate analysis and Poisson logistic regression analysis, respectively. Sex was excluded from this model, because no significant relationship with the community type was observed in the bivariate analysis.

***P < 0.001, **P < 0.01, *P < 0.05.

Abbreviations: CI, confidence interval; BMI; body mass index; DT, decayed teeth; DFT, decayed and filled teeth; PPD, periodontal pocket depth; BOP, bleeding on probing.

## References

[b1] Loesche W. J. Role of Streptococcus mutans in human dental decay. Microbiol Rev 50, 353–380 (1986). 3540569 10.1128/mr.50.4.353-380.1986 PMC373078

[b2] Socransky S. S., Haffajee A. D., Cugini M. A., Smith C. & Kent R. L. Jr. Microbial complexes in subgingival plaque. J Clin Periodontol 25, 134–144 (1998). 9495612 10.1111/j.1600-051x.1998.tb02419.x

[b3] Caufield P. W., Schon C. N., Saraithong P., Li Y. & Argimon S. Oral Lactobacilli and Dental Caries: A Model for Niche Adaptation in Humans. J Dent Res 94, 110S–118S (2015). 25758458 10.1177/0022034515576052 PMC4547204

[b4] Perez-Chaparro P. J. . Newly identified pathogens associated with periodontitis: a systematic review. J Dent Res 93, 846–858 (2014). 25074492 10.1177/0022034514542468 PMC4541103

[b5] Greenstein G. & Lamster I. Bacterial transmission in periodontal diseases: a critical review. J Periodontol 68, 421–431 (1997). 9182736 10.1902/jop.1997.68.5.421

[b6] Kaplan J. B. Biofilm dispersal: mechanisms, clinical implications, and potential therapeutic uses. J Dent Res 89, 205–218 (2010). 20139339 10.1177/0022034509359403 PMC3318030

[b7] Rasiah I. A., Wong L., Anderson S. A. & Sissons C. H. Variation in bacterial DGGE patterns from human saliva: over time, between individuals and in corresponding dental plaque microcosms. Arch Oral Biol 50, 779–787 (2005). 15970209 10.1016/j.archoralbio.2005.02.001

[b8] Stahringer S. S. . Nurture trumps nature in a longitudinal survey of salivary bacterial communities in twins from early adolescence to early adulthood. Genome Res 22, 2146–2152 (2012). 23064750 10.1101/gr.140608.112 PMC3483544

[b9] Yamanaka W. . Compositional stability of a salivary bacterial population against supragingival microbiota shift following periodontal therapy. PLoS One 7, e42806 (2012). 22916162 10.1371/journal.pone.0042806 PMC3420916

[b10] Zhou Y. . Biogeography of the ecosystems of the healthy human body. Genome Biol 14, R1 (2013). 23316946 10.1186/gb-2013-14-1-r1 PMC4054670

[b11] Goodson J. M., Groppo D., Halem S. & Carpino E. Is obesity an oral bacterial disease? J Dent Res 88, 519–523 (2009). 19587155 10.1177/0022034509338353 PMC2744897

[b12] Piombino P. . Saliva from obese individuals suppresses the release of aroma compounds from wine. PLoS One 9, e85611 (2014). 24465618 10.1371/journal.pone.0085611 PMC3899019

[b13] Yoshizawa J. M. . Salivary biomarkers: toward future clinical and diagnostic utilities. Clin Microbiol Rev 26, 781–791 (2013). 24092855 10.1128/CMR.00021-13 PMC3811231

[b14] Li K., Bihan M. & Methe B. A. Analyses of the stability and core taxonomic memberships of the human microbiome. PLoS One 8, e63139 (2013). 23671663 10.1371/journal.pone.0063139 PMC3646044

[b15] De Filippis F. . The same microbiota and a potentially discriminant metabolome in the saliva of omnivore, ovo-lacto-vegetarian and Vegan individuals. PLoS One 9, e112373 (2014). 25372853 10.1371/journal.pone.0112373 PMC4221475

[b16] Li J. . Comparative analysis of the human saliva microbiome from different climate zones: Alaska, Germany, and Africa. BMC Microbiol 14, 316 (2014). 25515234 10.1186/s12866-014-0316-1 PMC4272767

[b17] Huse S. M., Ye Y., Zhou Y. & Fodor A. A. A core human microbiome as viewed through 16S rRNA sequence clusters. PLoS One 7, e34242 (2012). 22719824 10.1371/journal.pone.0034242 PMC3374614

[b18] Hata J. . Secular trends in cardiovascular disease and its risk factors in Japanese: half-century data from the Hisayama Study (1961-2009). Circulation 128, 1198–1205 (2013). 23902756 10.1161/CIRCULATIONAHA.113.002424

[b19] Faith D. P. Conservation Evaluation and Phylogenetic Diversity. Biological Conservation 61, 1–10 (1992).

[b20] Costello E. K. . Bacterial community variation in human body habitats across space and time. Science 326, 1694–1697 (2009). 19892944 10.1126/science.1177486 PMC3602444

[b21] Kolenbrander P. E. . Communication among oral bacteria. Microbiol Mol Biol Rev 66, 486–505 (2002). 12209001 10.1128/MMBR.66.3.486-505.2002 PMC120797

[b22] Arumugam M. . Enterotypes of the human gut microbiome. Nature 473, 174–180 (2011). 21508958 10.1038/nature09944 PMC3728647

[b23] Nasidze I. . High diversity of the saliva microbiome in Batwa Pygmies. PLoS One 6, e23352 (2011). 21858083 10.1371/journal.pone.0023352 PMC3156759

[b24] Takeshita T. . Distinct composition of the oral indigenous microbiota in South Korean and Japanese adults. Sci Rep 4, 6990 (2014). 25384884 10.1038/srep06990 PMC4227031

[b25] Mager D. L., Ximenez-Fyvie L. A., Haffajee A. D. & Socransky S. S. Distribution of selected bacterial species on intraoral surfaces. J Clin Periodontol 30, 644–654 (2003). 12834503 10.1034/j.1600-051x.2003.00376.x

[b26] Segata N. . Composition of the adult digestive tract bacterial microbiome based on seven mouth surfaces, tonsils, throat and stool samples. Genome Biol 13, R42 (2012). 22698087 10.1186/gb-2012-13-6-r42 PMC3446314

[b27] Takeshita T. . Dental plaque development on a hydroxyapatite disk in young adults observed by using a barcoded pyrosequencing approach. Sci Rep 5, 8136 (2015). 25633431 10.1038/srep08136 PMC4311255

[b28] Ritz H. L. Microbial population shifts in developing human dental plaque. Arch Oral Biol 12, 1561–1568 (1967). 5237337 10.1016/0003-9969(67)90190-2

[b29] Chen H. . A Filifactor alocis-centered co-occurrence group associates with periodontitis across different oral habitats. Sci Rep 5, 9053 (2015). 25761675 10.1038/srep09053 PMC4356962

[b30] Griffen A. L. . Distinct and complex bacterial profiles in human periodontitis and health revealed by 16S pyrosequencing. ISME J 6, 1176–1185 (2012). 22170420 10.1038/ismej.2011.191 PMC3358035

[b31] Gross E. L. . Bacterial 16S sequence analysis of severe caries in young permanent teeth. J Clin Microbiol 48, 4121–4128 (2010). 20826648 10.1128/JCM.01232-10 PMC3020839

[b32] Albandar J. M. Global risk factors and risk indicators for periodontal diseases. Periodontol 2000 29, 177–206 (2002). 12102708 10.1034/j.1600-0757.2002.290109.x

[b33] Furuta M. . Relationship between periodontitis and hepatic abnormalities in young adults. Acta Odontol Scand 68, 27–33 (2010). 19878045 10.3109/00016350903291913

[b34] Suvan J., D’Aiuto F., Moles D. R., Petrie A. & Donos N. Association between overweight/obesity and periodontitis in adults. A systematic review. Obes Rev 12, e381–404 (2011). 21348914 10.1111/j.1467-789X.2010.00808.x

[b35] Takeshita T. . The ecological proportion of indigenous bacterial populations in saliva is correlated with oral health status. ISME J 3, 65–78 (2009). 18830275 10.1038/ismej.2008.91

[b36] Kononen E., Kanervo A., Takala A., Asikainen S. & Jousimies-Somer H. Establishment of oral anaerobes during the first year of life. J Dent Res 78, 1634–1639 (1999). 10520968 10.1177/00220345990780100801

[b37] Crielaard W. . Exploring the oral microbiota of children at various developmental stages of their dentition in the relation to their oral health. BMC Med Genomics 4, 22 (2011). 21371338 10.1186/1755-8794-4-22 PMC3058002

[b38] Xu X. . Oral cavity contains distinct niches with dynamic microbial communities. Environ Microbiol 17, 699–710 (2015). 24800728 10.1111/1462-2920.12502

[b39] Belstrom D. . Bacterial profiles of saliva in relation to diet, lifestyle factors, and socioeconomic status. J Oral Microbiol 6, doi: 10.3402/jom.v6.23609 (2014). PMC3974179 24765243

[b40] Mason M. R. . The subgingival microbiome of clinically healthy current and never smokers. ISME J 9, 268–272 (2015). 25012901 10.1038/ismej.2014.114 PMC4274424

[b41] Eren A. M., Borisy G. G., Huse S. M. & Mark Welch J. L. Oligotyping analysis of the human oral microbiome. Proc Natl Acad Sci USA 111, E2875–2884 (2014). 24965363 10.1073/pnas.1409644111 PMC4104879

[b42] Shimazaki Y. . Effectiveness of the salivary occult blood test as a screening method for periodontal status. J Periodontol 82, 581–587 (2011). 21043793 10.1902/jop.2010.100304

[b43] Pihlstrom B. L., Michalowicz B. S. & Johnson N. W. Periodontal diseases. Lancet 366, 1809–1820 (2005). 16298220 10.1016/S0140-6736(05)67728-8

[b44] Silness J. & Loe H. Periodontal Disease in Pregnancy. Ii. Correlation between Oral Hygiene and Periodontal Condtion. Acta Odontol Scand 22, 121–135 (1964). 14158464 10.3109/00016356408993968

[b45] Takeshita T., Nakano Y. & Yamashita Y. Improved accuracy in terminal restriction fragment length polymorphism phylogenetic analysis using a novel internal size standard definition. Oral Microbiol Immunol 22, 419–428 (2007). 17949346 10.1111/j.1399-302X.2007.00384.x

[b46] Edgar R. C. UPARSE: highly accurate OTU sequences from microbial amplicon reads. Nat Methods 10, 996–998 (2013). 23955772 10.1038/nmeth.2604

[b47] Haas B. J. . Chimeric 16S rRNA sequence formation and detection in Sanger and 454-pyrosequenced PCR amplicons. Genome Res 21, 494–504 (2011). 21212162 10.1101/gr.112730.110 PMC3044863

[b48] Caporaso J. G. . QIIME allows analysis of high-throughput community sequencing data. Nat Methods 7, 335–336 (2010). 20383131 10.1038/nmeth.f.303 PMC3156573

[b49] Chen T. . The Human Oral Microbiome Database: a web accessible resource for investigating oral microbe taxonomic and genomic information. Database (Oxford), baq013 (2010). 20624719 10.1093/database/baq013 PMC2911848

[b50] Price M. N., Dehal P. S. & Arkin A. P. FastTree: computing large minimum evolution trees with profiles instead of a distance matrix. Mol Biol Evol 26, 1641–1650 (2009). 19377059 10.1093/molbev/msp077 PMC2693737

