# Supplementary material for: Bacterial diversity in saliva and oral health-related conditions: the Hisayama Study
Source: Sci Rep. 2016 Feb 24;6:22164. doi: 10.1038/srep22164 (PMC4764907; doi:10.1038/srep22164)
Supplement: Supplementary Information [file srep22164-s1.pdf]

Bacterial diversity in saliva and oral health-related conditions: the Hisayama Study

Toru Takeshita[1], Shinya Kageyama[1], Michiko Furuta[1], Hidenori Tsuboi[1], Kenji Takeuchi[1], Yukie Shibata[1], Yoshihiro Shimazaki[2], Sumio Akifusa[3], Toshiharu Ninomiya[4], Yutaka Kiyohara[5], Yoshihisa Yamashita[1]*

[1]Section of Preventive and Public Health Dentistry, Division of Oral Health, Growth and Development, Kyushu University Faculty of Dental Science, Fukuoka, Japan, [2]Department of Preventive Dentistry and Dental Public Health, School of Dentistry, Aichi-Gakuin University, Nagoya, Japan, [3]Department of Oral Health Management, School of Oral Health Science, Kyushu Dental College, Kitakyushu, Japan, [4]Division of Research Management, Center for Cohort Studies, Graduate School of Medial Sciences, Kyushu University, Fukuoka, Japan, [5]Department of Environmental Medicine, Graduate School of Medical Sciences, Kyushu University, Fukuoka, Japan

Correspondence and requests for materials should be addressed to Y.Y.

Supplemental Figure Legend

Supplemental Figure 1. Rarefaction curves for a number of observed operational taxonomic units (OTUs) per sample. Mean number of observed OTUs almost reached a plateau by 5,000 sequence reads. The error bars indicate the standard deviation.

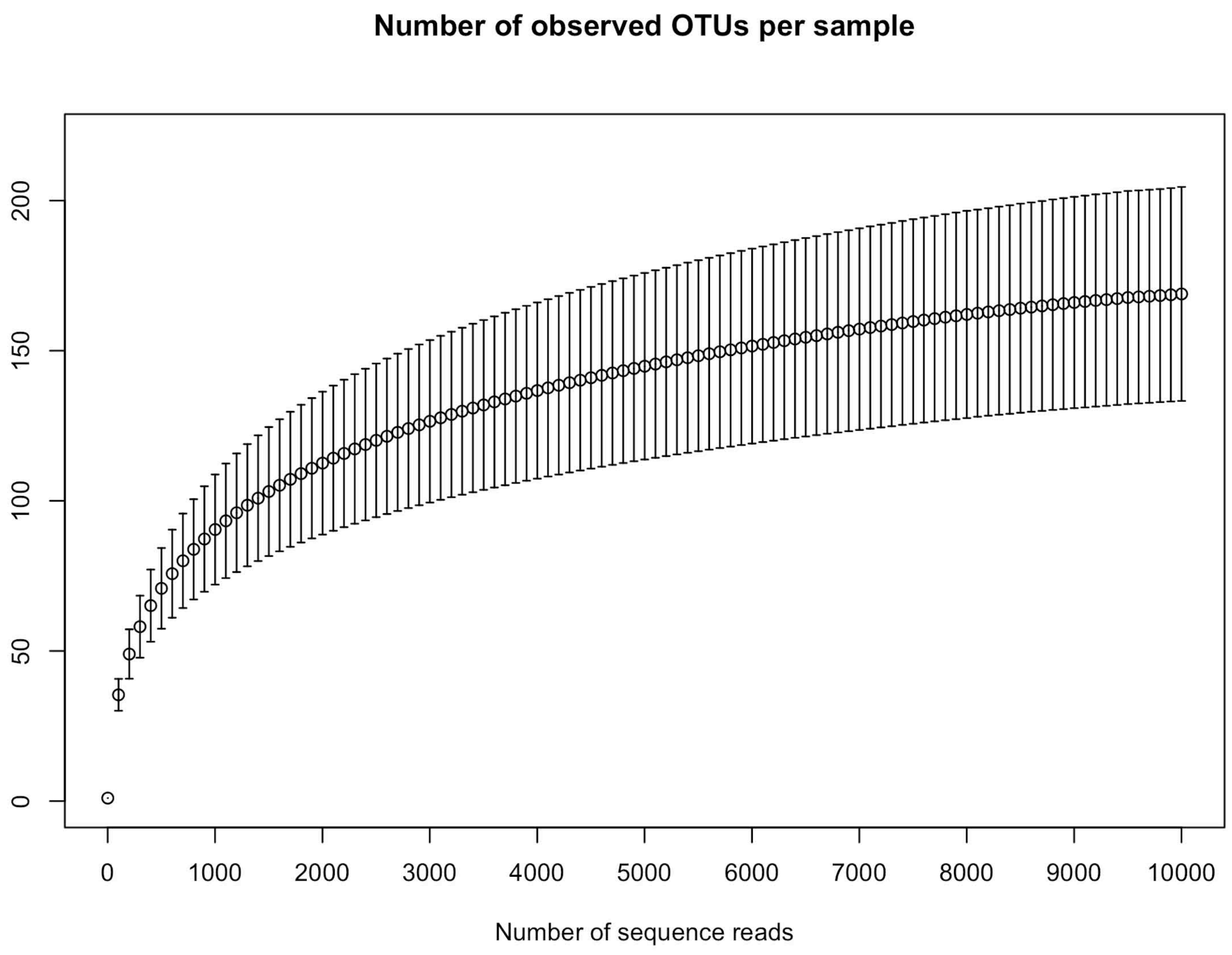
Number of observed OTUs per sample
0
50
100
150
200
0
1000
2000
3000
4000
5000
6000
7000
8000
9000
10000
Number of sequence reads

Supplemental Table

Table S1. Relative abundances of 22 dominant core operational taxonomic units (OTUs) in each community type.

	Community type			Bacterial phylum	Bacterial species
	Type I	Type II	*P* value[a]	corresponding to each OTU	corresponding to each OTU
OTU1	**2.9 ± 1.7**	1.5 ± 0.9	<0.001	*Firmicutes*	*Veillonella parvula* (161)
OTU2	0.7 ± 1.1	**4.3 ± 3.6**	<0.001	*Bacteroidetes*	*Porphyromonas* sp. (279)
OTU3	**2.2 ± 2.4**	0.4 ± 0.6	<0.001	*Bacteroidetes*	*Prevotella histicola* (298)
OTU4	**1.0 ± 1.2**	0.9 ± 1.1	0.009	*Bacteroidetes*	*Alloprevotella* sp. (308)
OTU5	**10.5 ± 6.7**	7.1 ± 4.7	<0.001	*Bacteroidetes*	*Prevotella melaninogenica* (469)
OTU6	**3.2 ± 1.8**	1.6 ± 1.0	<0.001	*Firmicutes*	*Veillonella atypica* (524)
OTU7	3.0 ± 2.0	3.1 ± 1.9	0.055	*Firmicutes*	*Granlicatella adiacens* (534)
OTU8	**1.9 ± 2.9**	1.0 ± 1.4	<0.001	*Actinobacteria*	*Rothia dentocariosa* (587)
OTU9	2.7 ± 3.1	**15.7 ± 8.7**	<0.001	*Proteobacteria*	*Neisseria flavescens* (610)
OTU10	4.1 ± 3.2	**4.7 ± 3.1**	<0.001	*Firmicutes*	*Streptococcus mitis* (677)
OTU11	**10.4 ± 8.3**	7.5 ± 5.5	<0.001	*Actinobacteria*	*Rothia mucilaginosa* (681)
OTU12	0.9 ± 1.4	**1.8 ± 1.8**	<0.001	*Proteobacteria*	*Haemophilus parainfluenzae* (718)
OTU13	**2.7 ± 2.2**	0.9 ± 0.9	<0.001	*Firmicutes*	*Streptococcus parasanguinis* (721)
OTU14	**5.1 ± 4.3**	1.6 ± 1.9	<0.001	*Firmicutes*	*Streptococcus salivarius* (755)
OTU15	1.4 ± 1.3	**2.1 ± 1.5**	<0.001	*Firmicutes*	*Gemella sanguinis* (757)
OTU16	**1.8 ± 2.6**	1.0 ± 1.6	<0.001	*Actinobacteria*	*Actinomyces graevenitzii* (866)
OTU17	0.8 ± 1.0	**1.3 ± 1.1**	<0.001	*Firmicutes*	Genus *Streptococcus*
OTU18	1.0 ± 1.3	**2.1 ± 1.6**	<0.001	*Firmicutes*	Genus *Streptococcus*
OTU19	**3.7 ± 3.3**	3.0 ± 2.8	<0.001	*Actinobacteria*	Genus *Actinomyces*
OTU20	**2.0 ± 2.4**	0.3 ± 0.5	<0.001	*Bacteroidetes*	Genus *Prevotella*
OTU21	3.5 ± 3.1	**3.8 ± 3.0**	0.020	*Firmicutes*	Genus *Streptococcus*
OTU22	**2.6 ± 2.4**	0.9 ± 1.0	<0.001	*Firmicutes*	Genus *Streptococcus*

[a]Significant differences between two community types were evaluated by using Student's t-test. The values that were significantly higher than the other community are shown in boldface.
